# Drama Therapy for Children and Adolescents with Psychosocial Problems: A Systemic Review on Effects, Means, Therapeutic Attitude, and Supposed Mechanisms of Change

**DOI:** 10.3390/children9091358

**Published:** 2022-09-06

**Authors:** Marij Berghs, Anna-Eva J. C. Prick, Constance Vissers, Susan van Hooren

**Affiliations:** 1 Royal Dutch Kentalis, 5271 GD Sint-Michielsgestel, The Netherlands; 2 KenVak, School of Arts Therapies, Zuyd University of Applied Science, 6419 DJ Heerlen, The Netherlands; 3 Department of Clinical Psychology, Faculty of Psychology, Open University of the Netherlands, 6419 AT Heerlen, The Netherlands; 4 Behavioural Science Institute, Radboud University, 6525 XZ Nijmegen, The Netherlands

**Keywords:** drama therapy, children, adolescents, youth, review, psychosocial problems, mechanisms of change, psychodrama

## Abstract

Drama therapy is applied to children and adolescents with psychosocial problems. Drama therapy is an experimental form of treatment which methodologically uses drama and theatre processes to achieve psychological growth. Although in clinical practice, drama therapy has been applied successfully, little is known about how and why drama therapy contributes to a decrease in psychosocial problems. A systematic narrative review was performed to obtain more insight into this issue. Eight databases were systematically searched. Ten out of 3742 studies were included, of which there were four random controlled trails, three non-controlled trials, and three pre-and post-test design studies. We identified the results, drama therapeutic means, attitude, and mechanism of change. Positive effects were found on overall psychosocial problems, internalizing and externalizing problems, social functioning, coping and regulation processes, social identity, and cognitive development. An adaptive approach was mentioned as the therapeutic attitude. The means established contribute to a dramatic reality, which triggers the mechanisms of change. These are processes that arise during treatment and which facilitate therapeutic change. We found ten supposed mechanisms of change to be frequently used in all studies. No direct relations were found between the results, drama therapeutic attitude, means, and mechanisms of change.

## 1. Introduction

Psychosocial problems consist of a combination of emotional, behavioral, and social problems [1,2]. One out of five children and one out of seven adolescents suffer from psychosocial problems, including mental disorders [3,4,5,6]. The mean global coverage of prevalence for mental disorders in children aged 5–17 years was 6.7% in 2016, subdivided into conduct disorder (5.0%), attention deficit/hyperactivity disorder (5.5%), autism spectrum disorders (16.1%), eating disorders (4.4%), depression (6.2%), and anxiety (3.2%) [7]. As an expression of dysfunction related to psychosocial problems, a distinction can be made between internalizing and externalizing problems. Internalizing problems concern emotional problems that focus inward, such as depression, (social) fears, withdrawn behavior, and psychosomatic complaints. Externalizing problems concern behaviors that are more outwardly directed, such as hyperactivity, aggressive behavior, and attention problems [8,9]. Children and adolescents who suffer from psychosocial problems are more likely to be a victim or a bullying perpetrator [10,11,12], experience lower academic performance [13,14], and have an increased risk of suicide [3,13,15,16]. Failure to identify and treat psychosocial problems in time increases the risk of problems in the future [17], for example, of physical disorders [18]. These problems have economic consequences which create additional costs for the society [19,20]. Many psychosocial problems in children and adolescents are not recognized and treated in time [21]. Addressing these problems at early age is necessary to prevent them from getting worse [22]. 

The most common treatment for children and adolescents with psychosocial problems are cognitive behavior therapy focusing on cognitive behavior, psycho-education, emotion regulation, communication, interpersonal skills, or parent training [23,24,25,26,27]. Some studies suggest that cognitive-behavioral therapy is less appropriate for young children and for children and adolescents who have difficulty expressing themselves verbally [28,29,30,31,32,33]. Activating strategies, such as role-playing, are emphasized as effective elements in treatment for these children and adolescents. In particular, role play is seen as important for modeling behavior, to expose fears, and as an opportunity to develop coping skills [27]. Activating strategies, and role play, in particular, are important elements in drama therapy to treat psychosocial problems in children and adolescents [31,34,35]. 

Drama therapy is an experiential form of psychotherapy which methodologically consists of drama and theatre processes, fictional reality created by a wide range of verbal and non-verbal dramatic techniques aimed to achieve psychological growth and change within a therapeutic relationship [36,37,38]. Drama therapy is one of the creative arts therapies (together with psychodrama, art therapy, dance and movement therapy, music therapy, and bibliotherapy). In drama therapy, drama and theater processes are influenced by and based on different psychological perspectives such as psychodynamic, cognitive behavioral therapy, attachment theory, and developmental psychology, client-centered therapy, or narrative theory [30,39,40,41,42,43]. Drama therapy is considered suitable for children because of the underlying play. Dramatic play is seen as one of the core processes in drama therapy [39,44,45,46,47]. Dramatic play gives children the opportunity to express (non-)verbally, gain control of their thoughts and feelings, and understand others. A variety of means, i.e., forms and techniques, are used in drama therapy, such as role-play, storytelling, puppet play, and theater games. These are aimed at creating a playspace where children can play in a fictional world. Although playing takes place in a dramatic (“as if”) reality, behavior, thoughts and feelings can be real at the same time. Hence, there is both a distance and a connection between play and daily life [39,46,47,48]. 

Attunement within the therapeutic relationships is important. The drama therapist adaptively matches the drama therapeutic means (e.g., drama role, themes) to the needs, expression, and wishes of the client [49,50,51].

In clinical practice, drama therapy is successfully applied by drama therapists using a variety of drama therapeutic approaches and theories based on good practice, theoretical insights, and intuition [30,49,52]. In a qualitative study, drama therapists reported several effects of drama therapy in children and adolescents, such as improvement of social skills, regulation of emotions, better child and adult relationship, increased assertiveness and self-expression, and more resilient responses to bereavement, separation, and loss [53]. These outcomes are important effects that may promote self-esteem that buffers the negative effect of stressful life events in adolescence [54]. Drama therapy experts assume that drama therapy is used to promote understanding of one’s own and others’ behavior in terms of mental states (mentalization) [55,56,57], executive functions [58,59], working memory [60,61], and resilience [39,62,63]. Most studies on the effects of drama therapy in children and adolescents are based on expert opinions reflecting on their clinical work. An overview of effects based on empirical studies using cohort studies and (randomized) controlled trials is still lacking.

Besides the effects of drama therapy on children and adolescents, little is known about what and how drama therapy leads to a positive change in psychosocial problems of children and adolescents [27,34,64]. There is a growing interest in insights into the effectiveness of drama therapy works and which processes contribute to changes of the client’s wellbeing. These processes are called mechanisms of change, referring to processes that arise during the treatment that facilitates the therapeutic change [65,66]. A few mechanisms of change are described in drama therapy. For example, drama therapists and adult clients describe the importance of a positive therapeutic relationship, working within a safe distance, being actively involved in the therapy, and having physical experiences that facilitate the development of new awareness and language skills through which clients can communicate to themselves and others [67]. 

The existing body of literature provides a first insight into the effects of drama therapy and how this may lead to a decrease in psychosocial problems in children and adolescents. However, overarching research specifically addressing the effectiveness of the different means of drama therapy on positive change is lacking. Therefore, an overview of the literature is necessary. The aim of this systematic review was first to identify the effects of drama therapy for children and adolescents and second to gain more insight into what kinds of drama therapeutic means, therapeutic attitude, and specific drama therapeutic mechanism of change are related to these effects. 

## 2. Methods

### 2.1. Study Design

A systematic narrative review was performed for study identification, selection, data extraction, and quality appraisal, using the guidelines from the Cochrane Collaboration [68].

### 2.2. Search

**Figure 1:**
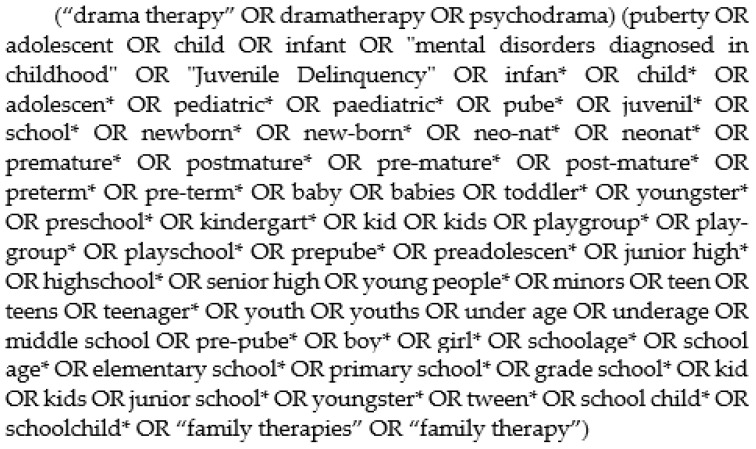
Search terms.

We systematically searched for articles. The following database and journals were searched: PsychINFO (EBSCO), Pub Med, ScienceDirect, Medline, Cinahl, Academic Search, Google Scholar and Drama Therapy Review. The search terms for all databases were (“drama therapy” OR dramatherapy) AND (child* OR youth OR adolescent). For all search terms, see Figure 1 Search terms. The literature study covers a period up to 1 September 2020. This study followed the guidelines of Preferred Reporting Items for Systematic Reviews and Meta-Analyses (PRISMA) [69].

### 2.3. In- and Exclusion Criteria

Studies on the effects of drama therapy for children and adolescents until 18 years were included. Regarding study design, we included randomized controlled trials (RCT’s), non-controlled trials (CCT’s), and pre- and post-test designs. Furthermore, we only included studies in which drama therapy was applied by a drama therapist. Only articles and theses written in English were included. We excluded studies in which the intervention was applied by another profession than a drama therapist, e.g., teacher or a nurse.

### 2.4. Selection of Studies

In two phases, the articles were selected based on the inclusion criteria using the web application Rayyan [70]. In the first phase, the researcher independently selected the articles based on title and abstract in four pairs. In the second phase, eligible articles were selected based on reading the full text. The first author was contacted when insufficient information was provided on our inclusion criteria. If there was doubt or disagreement in selecting a study, it was solved by discussion until consensus was reached.

### 2.5. Quality Assessment of Individual Studies

We coded whether the study was strong, moderate or weak with the “Quality Assessment Tool for Quantitative Studies” [71]. By providing a comprehensive and structured assessment of the concept of study quality, this tool assesses the quality of a study [72] The content and construct validity of the “Quality Assessment Tool for Quantitative Studies” has been reported [73,74]. The quality of the studies was assessed independently by four raters in a group of three duos, and then scores were compared. In case of disagreement, it was solved by discussion until consensus was reached.

### 2.6. Data Collection Process and Analysis

The data following were extracted from each study on: formal characteristic of included studies, i.e., first author/year, design/time points, quality assessment rate, study population, *n* = (treated/control), type (group or individual or both), frequency, duration, and control invention/care as usual (see Table 1), and results and description of effects drama therapy intervention, i.e., psychosocial outcome domain/measure, results, effect sizes (see Table 2), and characteristics of drama therapy interventions, i.e., goal of the study, intervention, therapist attitude, and drama therapeutic means and supposed mechanism of change of the intervention (see Table 3). When information was missing, we emailed the corresponding author of the study with a request for more information. A content analysis was performed on the effects of the interventions, the means, therapist attitude, and the described mechanisms of change [28]. A narrative approach was applied to synthesize the findings.

## 3. Results

This section may be divided by subheadings. It should provide a concise and precise description of the experimental results, their interpretation, as well as the experimental conclusions that can be drawn.

### 3.1. Study Selection

**Figure 2:**
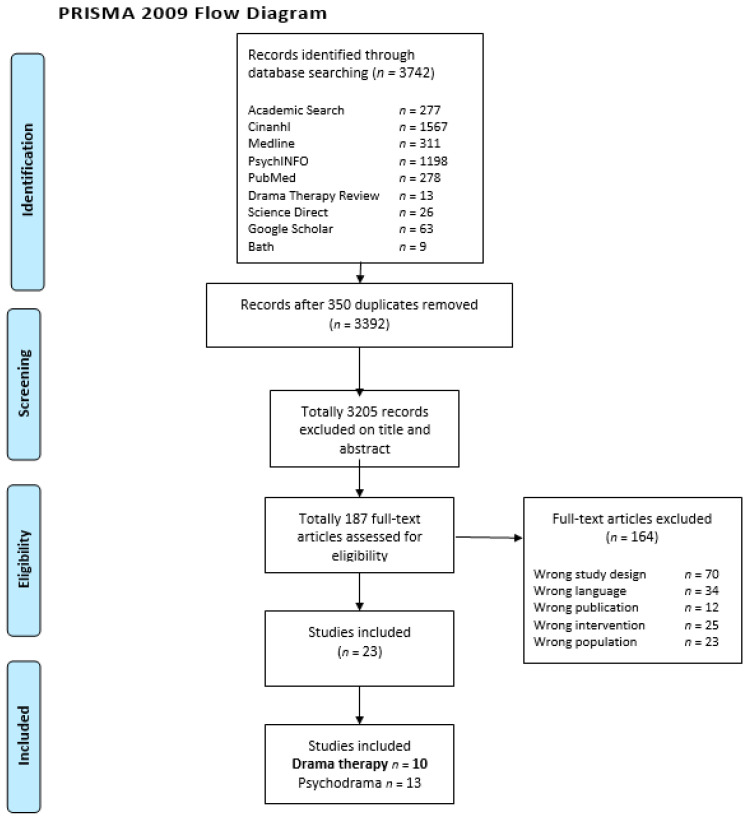
Flow chart of the search results.

The search resulted in 3742 studies on drama therapy and psychodrama (as a part of a wider review research) for children with psychosocial problems. In the first search, 3369 articles were found (June 2018) and 373 articles in the second search (September 2020). We removed 350 duplicate articles and excluded 3205 articles based on title and abstract. A total of 187 articles were selected for full text. Of these, we excluded 164 studies, 70 had the wrong study design, 34 studies were written in the wrong language, 12 articles had the wrong publication type such as a book, 25 studies had the wrong intervention, and 23 studies consisted of the wrong population. In total, ten studies on drama therapy were included. See Figure 2, flow chart of the search results, for a flow diagram of article eligibility for inclusion in the current review.

### 3.2. Quality of the Studies

Of the ten included studies, two studies were evaluated having a high quality [83,84], three studies a moderate quality [75,76,79], and five studies a weak quality [77,78,80,81,82]. The studies evaluated as strong were both RCT studies. Of the studies with moderate quality, one had a CCT design [75] and two a pre- and post-test design [76,79]. Of the five studies having weak quality, two had a RCT design [80,81], two a CCT design [77,78], and one a pre- and post-test design [82]. See Table 4, quality of the studies.

### 3.3. General Study Characteristics

There were four studies with an RCT design [80,81,83,84], three studies with a CCT design [75,77,78], and three studies with a pre- and post-test design [76,79,82]. The control group did not receive intervention [75,77,81,83,84], care as usual [78] or other interventions (psychotherapy or recreation activities) [80]. In total, there were 334 participants involved in the included studies of which there were 178 participants in the experimental group, 143 participants in the control group and 22 participants in the non-controlled design studies. Sample sizes varied from *n* = 5 to *n* = 123. See Table 1, formal characteristics of included studies.

### 3.4. Clients Characteristics

The study population consisted of emotionally disturbed children [80], children with a developmental disorder such as high function autism [76], children who coped with anxiety such as social anxiety [75], children who were shy and maladjusted [81], girls who had been sexually abused [82] and (newly arrived) immigrants and refugees [83,84]. In addition, one study included adolescents with several problems, i.e., a specific mental disorder, attention deficit hyperactivity disorder with aggression regulation problems, or a moderate-to-high recidivism risk [78]. One study did not provide a description of the population [77]. The age range of the total population of the studies included was 3.5 to 19 years. One study in total focused on 12 children in the age of 3–5 years [77], one study involved 12 children in the age of 7–8 [80], two studies in total focused on 31 children in the age of 10–14 [75,76,79], three studies focused on a total of 183 adolescents in the age of 12–18 years [82,84], one study focused on 91 adolescents of 16–19 years [78], and one study involved a broader age range of 5 participants of 9–16 years [81]. Four settings were related to school: day nursery [77], elementary [75], high school [76,83], and school psychological service [81]. Three settings were specialized centers: a secure juvenile justice institution [78], a specified social service center [76], and an outpatient treatment center [80]. Two settings were especially organized for the studies [71,74,79,82]. See Table 1, formal characteristics of included studies.

### 3.5. Drama Therapy Characteristics

In eight studies, drama therapy was the main treatment. In two studies, drama therapy was part of behavior therapy [81] or responsive aggression regulation therapy [78]. The frequency of the sessions in eight studies was once per week [76,78,80,82,83,84], in one study twice per week [75], in another six successive weekdays [77], and in one study four days per week [79]. One study did not mention the frequency of the sessions [81]. The duration of the drama therapy was from 6 to 21 sessions. Most studies had a duration of 6–14 sessions [75,77,78,79,82,83,84], two studies of 20–21 sessions [76,80], and one study did not mention the duration [81]. The length of the session was 60 min to 4–5 h. The length of the sessions in most studies equaled 60–90 min [76,77,78,80,83,84], and other studies reported a length of 2 to 5 h [75,79,82]. One study did not mention the length of the session [81]. Drama therapy was group based [75,76,79,80,82,83,84] or a combination of individual and group drama therapy [77,78,81]. Overall, we found that the drama therapy interventions were not consistently described. Two studies described the method of which the intervention was based on: Emunah’s Integrative Five-phase Model [75], Augusto Boal’s forum theatre, and Jonathan Fox’s playback theater [83]. One study described the elements of drama therapy: dramatic projection, dramatic reality, role-playing, and storytelling [76]. Two studies mentioned the drama therapy techniques, such as imagination, roleplaying games, and exercises where adolescents were stimulated to adopt new roles [78,79,81]. Two studies described the goal of the drama therapy intervention [80]. Three studies gave a description of the structure of each session [77,82,84]. See Table 1, formal characteristics of included studies; and Table 3, characteristics of drama therapy interventions.

### 3.6. Outcomes

Data were collected via self-reports [75,76,78,79,81,82,83,84], parents’ reports [76,80], teachers’ or staff members’ reports [78,83], or by tests (IQ, (neuro)psychological tests) [77,78,80,81]. Seven studies used current and valid questionnaires/measurements [75,76,77,78,79,82,83,84]. One or more questionnaires/measuring instruments of four studies were outdated [79,80,81,82]. Five studies did not use existing questionnaires and made use of their own developed reports/measuring instruments [77,78,79,80,81]. The results of one study [77] and the results of the Rorschah Index of Repressive Style test [80] could not be interpreted for meaning and therefore were not included in the analyses of the outcomes. See Table 3, results and description of effects drama therapy intervention.

### 3.7. Outcome Psychosocial Problems

The included studies focused on a range of outcomes. We categorized the outcome in seven categories, i.e., overall psychosocial problems, internalizing problems, externalizing problems, social functioning, coping and regulation processes, identity, and cognitive development.

#### 3.7.1. Overall Psychosocial Problems

Four studies focused on overall psychosocial problems [76,79,83]. This category consists of outcomes on overall psychosocial problems, problem behavior related to autism, and effect as an underlying concept for emotional functioning. The studies involved six children in the age of 10–12 years [76] and 199 adolescents in the age of 12–18 [79,83,84]. Two studies had a RCT design [83,84], and the other two studies had a pre- and post-test design [76,79]. One study examined effects on psychosocial problems reported by the adolescents and their teachers [83]. The study showed differences between psychosocial problems reported by the adolescent versus the teacher: a decrease in overall psychosocial problems was found reported by adolescents, while no effect was found reported by teachers [77]. A positive effect reported by adolescents was also seen in another study examining effects on psychosocial problems [84]. One study examined autism problem behavior, both reported by the parents as well as by the students themselves. No effect was found on the autism problem behavior after the intervention [76]. There was one study examining the effects of intervention on negative and positive affect. An increase in positive affect was found, but no effect was found for negative affect [79].

#### 3.7.2. Internalizing Problems

Six studies focused on the effects of drama therapy interventions on internalizing problems [75,76,79,81,82,83]. The category internalizing problems consisted of outcomes regarding anxiety, depression, (di)stress and posttraumatic stress, timidity, obsessive compulsive disorder, interpersonal sensitivity, and somatization. The studies involved 164 children in the age of 9–18 years. Two of the studies had a RCT design [81,83], one study had a CCT design [75], and three studies had a pre- and post-test design [76,79,82]. One study examining the effect of drama therapy interventions on internalizing problem behavior rated by the parents and students did not show an effect on this outcome [76]. Two studies examined the effect on anxiety [79,82]. The results of one study showed a decrease in anxiety [79], and the results of the other study did not show any effect on anxiety. Two studies examined effects on specific anxieties, i.e., social anxiety [75] and phobic anxiety [82]. Only a decrease was seen for social anxiety. Two studies examined the effects on depression. Results of both studies showed a positive effect on this outcome [79,82]. Two studies examined the effects on stress, i.e., distress [83], and symptoms of posttraumatic stress [79]. The results of both studies showed a decrease in distress rated by the students, and in one study, there was also a decrease in symptoms of posttraumatic stress, while the results rated by the teachers did not show an effect on distress. Other studies examining the effect on psychopathology symptoms showed a decrease in symptoms of psychotic thinking [82] and in severe timidity [81], while there was no effect on somatization, paranoid ideation, interpersonal sensitivity, and obsessive compulsive disorder [82].

#### 3.7.3. Externalizing Problems

Three studies focused on the effect of the drama therapy interventions on externalizing problems [76,78,82]. This category consisted of outcomes on overall externalizing problem behavior, impulsivity, hyperactivity, (in)attention, assertiveness, hostility, violent recidivism risk, and the number of registered incidents. The studies involved six children in the age of 10–12 years [76], five adolescents in the age of 12–18 [82], and 91 adolescents in the age of 16–19 years [78]. One study had a CCT design [78], and the other studies had a pre- and post-test design [76,82]. One study examined externalizing behavior, hyperactivity, and inattention, both self-rated as well as rated by their parents. No effect was found for externalizing behavior rated by the students. However, parents’ ratings showed a decrease in externalizing problems behavior. In addition, both student and parents reported a decrease in hyperactivity and inattention [76]. Another study examining inattention and impulsivity showed a decrease in symptoms on both inattention and impulsivity [78]. One study examined results on hostility [82], and one study examined assertiveness and violent recidivism risk behavior [78]. The results of these studies showed a decrease in hostility and violent recidivism risk behavior and an increase in assertiveness, but there was no increase in the number of registered incidents [78].

#### 3.7.4. Social Functioning

Three studies [76,82,83] focused on the effect of drama therapy intervention on social functioning. This category consisted of outcomes related to social skills, more specially the perception of the students and teachers regarding the extent to which psychosocial problems interfered with home life, friendship, leisure activities, the outcome on self-esteem in social behavior and the satisfaction with social support. The studies involved 134 children in the age of 11–18 years. One of the studies had a RCT design [83], and the other studies had a pre- and post-test design [76,82]. One study examined effects on overall social skills rated by the children and by their parents. The results showed a positive effect on overall social skills rated by the parents, while the results rated by the children did not show any effects of intervention on overall social skills. Results regarding more specific socials skills, such as communication, cooperation, responsibility, empathy and self-control, rated by the children and by their parents, did not show any effects. However, the amount of engagement rated by the parents showed an increase after the intervention [76]. One study examined the effects on satisfaction with social support and social desirability behavior; no differences were found after the interventions [82]. Another study examined to what extent the psychosocial problems interfere with friendship, with home life, and with leisure activities from the perception of students and their teachers. A positive effect was observed when the scores of the students were analyzed, while no effects were found for the scores of the teachers [83].

#### 3.7.5. Coping and Regulation Processes

Three studies focused on the effects of the drama therapy interventions on coping and regulation processes [76,78,82]. This category consisted of coping skills, cognitive distortions, self-control and regulation processes. The studies involved 102 children in the age of 10–19 years. Two of the studies had a pre- and post-test design [76,82], and one study had a CCT design [78]. Two studies examined the effects on self-control. The results of one study showed a positive effect on self-control rated by the adolescents and their by mentors [78]. However, results of the other study did not show any effects on self-control rated by students and by their parents [76]. One study examined dealing with anger, assertiveness, distrust, and coping skills (problem solving, palliative coping, social support, reassuring thought, stress, and poor coping) rated by the juvenile and rated by the mentors. These results showed a positive effect on dealing with anger, assertiveness, and on coping skills (problem solving, palliative coping, social support, reassuring thought, stress, and poor coping). The same study examined the effect on cognitive distortions (aggression, justification, physical aggression, oppositional behavior, sub assertive behavior, and negative attitude) and found a decrease in cognitive distortion, but did not find an effect on negative attitude [78]. In addition, an increase in motivation for treatment was found. One study examined the effects on the attribution style in good and bad situations, but no effect was found [82].

#### 3.7.6. Social Identity

Three studies focused on the effects of drama therapy interventions on social identity [80,82,83]. This consisted of attitude change and self-esteem. The studies involved 12 children in the age of 7–8 years [80] and 128 adolescents in the age of 12–18 [82,83]. Two of the studies had a RCT design [80,83], and one had a pre- and post-test design [82]. One study examined the effect on attitude change. The results showed a positive effect on the way the children evaluate themselves and other children. Furthermore, the results showed a positive effect on the amount of potency the children saw for themselves, other children and adults. There was no effect on attitude change regarding activity, sharing, imagination, and feelings [80]. In the study examining the effect on self-esteem, no effect was found [82,83].

#### 3.7.7. Cognitive Development

Four studies focused on the effects of drama therapy intervention on cognitive development [78,80,81,83]. This category consisted of a subset of cognitive functions and abilities: language skills, academic performance, attention deficit and cognitive structure. The studies involved 12 children in the age of 7–8 [80] and 229 adolescents in the age of 9–19 years [78,81,83]. Three of the studies had a RCT design [80,81,83], and the other study had a CCT design [78]. Two studies examined the effect on academic performance in mathematics and one study on reading and spelling. The results in one study showed a positive effect on mathematics [83], and the results of the other study did not show effects on mathematics, reading or spelling [81]. Two studies examined the effect on language development in terms of oral expression [80,83], and results showed an increase in oral expression. One study examined the effect on attention deficit as a neuropsychological outcome. The results showed a decrease in attention deficit [78]. One study examined the effect on the perception of the extent to which the impairment interfered with classroom learning. The results rated by the students and by the teachers did not show an effect on the perception of the extent to which the impairment interfered with classroom learning [83].

### 3.8. Outcome Drama Therapy Characteristics

To gain more insight into the effects of drama therapy treatment on psychosocial problems in children and adolescents, we analyzed the drama therapeutic intervention, means, therapeutic attitude, and mechanism of change.

#### 3.8.1. Drama Therapeutic Means

The drama therapy means are the forms and techniques of drama therapy that were applied during the drama therapy sessions. Two studies mentioned *dramatic reality* [76] as a means where children and adolescents create a fictional reality based on their imagination [76] or based on personal stories [84] and dramatic reality as a projective technique where the children and adolescents project inner feelings on dramatic representations [84].

Three studies applied projection as a means in different forms such as *dramatic projection* [75], *projective techniques* [79], *symbolic play as a projective technique* [82] where the children and adolescents project unconscious inner feelings at a safe distance [76,82] and verbalize how they felt [79]. *Role playing* was also mentioned as a projective technique in one study [76]. This is where the children had the opportunity to empathize with the role and project their ideas about how their feelings.

*Storytelling* [76], *symbolic play* [83] and *pairs techniques* [83] were also mentioned as a reflective technique where the adolescents reflect on their points of view and feelings [83]. *Storytelling* was also mentioned in three other studies. One study used storytelling as a technique to create a symbolic and safe distance from reality [82]. Another study mentioned storytelling as a means that was used to share strong emotions and subsequent relief [83]. One study used storytelling focusing on group play and social interaction. In this study, *movement*, *voice, role play*, and pantomime were used focusing on group play and social interaction [75].

Four studies [79,80,83,84] used *acting out* personal stories as a means to transform these stories into alternative scenarios developed by group members [83] or to express feelings [80,84].

Three studies mentioned *improvisation* [79,80,82] as a means where the children and adolescents adopt new roles, and explore spontaneity [79], express and play out feelings [80], and can play a variety of roles attuned to their needs and requests [82].

One study used *role-playing games* to practice perspective-taking exercises [78]. One study mentioned *theatrical exercises* as a means to transform the experience of adversity [84]. *Playing a role* was mentioned by one study as a means to express the inner characteristics of the role in a way that can be understood by others [80].

#### 3.8.2. Drama Therapeutic Attitude

Three studies reported the therapeutic attitude [76,79,82]. All of them described an adaptive approach where the drama therapists created opportunities to cooperate, build cohesion, share feelings and where the children and adolescents are accepted as being of unconditional worth. One of the studies mentioned specifically that the adaptive approach was based on the view of Carls Rogers [82].

#### 3.8.3. Supposed Mechanisms of Change

We categorized the mechanisms of change into two categories: specific drama therapeutic mechanisms of change and general mechanisms of change.

##### Specific Mechanisms of Change

Nine categories were identified reflecting specific mechanisms of change which contribute to the effectiveness of the drama therapy intervention. The first category was related to the process where *expression is stimulated in drama therapy*. These processes concern those that stimulate participants to express their own ideas [76], emotions [76,79,83], experiences [76], thoughts [76,82], internal states in verbal terms [79,80], verbally and non-verbally in a role [80,83], and their identities [82]. The second category concerned the process of sharing experiences and feelings [72,76,79,83], emotions of oneself and others [79], and personal stories [84]. The third category was the process that allows participants to *gain experiences in the drama therapy*. Experiences that were mentioned are related to positive relations [76], social connections [76], fun and playfulness [76,82], getting closer to each other [82], acting out ideas and feelings [82], control in the role-play [82], and recreating and experiencing life situations [75]. The fourth category concerned processes in the drama therapy where participants *become aware* of their vulnerability and psychological issues [76], new identities [76], life roles [79], and ideas and feelings (which associate with key experiences) [83]. The fifth category was the *process of reflection* on experiences [76,79,82], feelings [76,79,80], different points of view [78,83], oneself [76,79,80] and others [76,79,80] in the drama therapy. The sixth category was the *process of embodying* the personas [76] and emotional experience [79]. Embodiment is considered as a process to internalize new roles [79] in the drama therapy. The seventh category was the process in which participants *witness others in the drama therapy* [80]. The eighth category is the processes in which participants *gain self-control in the drama therapy* by becoming more active during their own treatment [68] and gain a sense of agency [83]. The ninth category is the process in which participants are *stimulated to be creative in the drama therapy* [76,82] and are *stimulated to use their imagination* [76,79].

##### General Mechanisms of Change

One general category of mechanism of change was found. This is drama therapy as a group process where participants *share* experiences [76,79], feelings [72,76,79], emotions [79], thoughts about experiences [82], strong emotions and subsequent relief [83] and their stories [84].

## 4. Discussion

The aim of this systematic review was to gain insight into the effects of drama therapy on psychosocial problems in children and adolescents. To this aim, the means and the general and specific mechanisms of change were identified that contribute to a decrease in psychosocial problems. This review showed that studies focused on a variety of psychosocial problems and age groups. In addition, drama therapy was applied as both curative and preventive. Most drama therapy interventions described in the studies were group based, in which there is room to pay attention to individual therapeutic goals. Furthermore, the content, duration and timing of the treatment varied from 6 to 21 sessions. This wide range of both client and drama therapy characteristics showed that drama therapy is applied within a diversity of target groups with psychosocial problems at all ages (3.5–19 years), both individually and in a group, within different (specialized) settings, both preventive and curative.

Results of this review showed that drama therapy can contribute to a decrease in psychosocial problems in children and adolescents. Positive effects of drama therapy were found for overall psychosocial problems and positive affect. Regarding internalizing problems, a decrease in depressive symptoms and symptoms of posttraumatic stress was observed. We also found a decrease in distress reported from the perspective of the children, while this was not reported by teachers. Reduction in anxiety symptoms was less consistently demonstrated. In one study [82], no positive effects were shown, while in two other studies, a reduction in anxiety [79] and specifically social anxiety [75] was shown after drama therapy. Regarding externalizing problems, we found a decrease in externalizing problem behavior reported by parents, while this was not seen from the perspective of the children [76]. In addition, drama therapy resulted in a decrease in inattention in two studies; more specifically, positive effects were seen for hyperactivity [76] and impulsivity [78]. In one study [78], in which drama therapy was a part of the larger treatment program, we also found a decrease in aggressive behavior in the form of hostility, violent recidivism risk behavior, and an increase in assertiveness. It is unclear to what extent drama therapy contributed to these effects.

Positive effects of drama therapy on social functioning were not found consistently. In one large-scale (*n* = 123) study [83], adolescents showed a decrease in the extent to which the symptoms impacted their social functioning in terms of their friendships, family life, and leisure activities, while this was not reported by the teachers. Regarding social identity, one small study [80] had suggested promising results, since drama therapy appears to result in a change in attitude of the children or adolescents toward themselves and how they evaluate themselves and others. No positive effects were found on self-esteem in this review. This is remarkable, since in clinical practice, drama therapy is often applied to increase self-esteem [46,47,49,50,52,53,110,111,112,113]. This discrepancy between clinical practice and the results of the included studies can be explained by the fact that both studies investigated brief therapies that were not directly aimed at enhancing self-esteem. In addition, in clinical practice, drama therapy is often applied to learn new coping skills and regulate behavior. In our review, only one study [78] found positive effects on coping skills and regulation processes, while this was not confirmed in two other studies [76,82]. In this study, drama therapy was part of a broader treatment, and therefore, it is unclear to what extent drama therapy contributed to these effects. Finally, four studies examined effects on cognitive development. Results showed better performance on mathematics, oral expression, and a reduction in attention deficit. This can be considered an indirect effect, since drama therapy interventions were not targeting these school abilities. Possibly, drama therapy improves prerequisites for learning, such as feeling safe, less anxious and less distracted, which has a positive impact on school abilities.

Some of the positive effects were dependent on perspective, i.e., whether the child/adolescent or the parent/teacher filled out the questionnaire. Overall, parents and teachers reported positive effects on behavior (i.e., fewer externalizing problems, and improved social functioning, and social identity), whereas these positive changes were not found when children or adolescents were asked. Furthermore, children and adolescents often reported positive effects when asked about their inner states, such as internalizing problems, whereas these positive effects were not found when parents/teachers filled out the questionnaire. It could be that explicit or externalizing behavior is better and earlier observed from an external perspective, whereas this is not the case for internalizing behavioral problems. In addition, it is not clear how parents and teachers were involved in the treatments.

Since not all studies systematically described the means, therapeutic attitude or supposed mechanisms of change in the drama therapy intervention, a narrative approach was applied to synthesize the findings in the literature. These results showed a broad variety of drama therapeutic means that were used in drama therapy. These means ranged from means focusing on group play and social interactions such as storytelling, movement, voice, role play, pantomime [75] or theatrical exercises [84] to projective techniques such as dramatic reality [76], dramatic projection [76] and symbolic play [82], and reflective techniques, for example, storytelling [76] and symbolic play [83]. Some means have more than one purpose, e.g., to reflect as well as to project. In addition, in some means, the exploration, expression, and experience of new roles were central. Examples of these means are acting out [80,83,84], improvisation [79], and playing a role [80]. Finally, in some means, perspective taking was emphasized, such as role playing [78].

The means are all forms or techniques that were applied during the drama therapy sessions and which contributed to the creation of dramatic play and eventually a dramatic reality. During dramatic play, clients are encouraged to respond spontaneously and to explore, create and play different characters with different feelings and behaviors. This takes place in a “playspace”, where clients can act and play at a safe distance from experiences in daily life [62]. This is where dramatic play feels “real”, but not overwhelming, as may be the case in real life. In such moments, it becomes a dramatic reality [48]. Experience in the dramatic reality may trigger a change. Dramatic projection is considered as one of the core processes of drama therapy [46,114]. Dramatic projections are techniques used by drama therapists to translate clients’ feelings and inner experiences from real life into dramatic representations so that these feelings can be externalized and expressed [46,114,115,116]. In addition, reflective techniques are important in drama therapy. The clients can reflect on different perceptions and perspectives in play in relation to everyday responses. This will be a crucial step to explore the expression of inner states into more appropriate responses in dramatic reality, by means of symbolic play, storytelling and/or pair techniques applied by the drama therapist [46,47,117,118]. The means found in this review are considered some of the basic means of drama therapy which prompt children and adolescents to explore feelings, behavior, and wishes in different forms of dramatic reality [62,116,119,120]. In this respect, there is a triangular relationship between the client, drama and theatre processes, and the drama therapist [43,47,121]. In all studies, the drama therapeutic means were considered as a third dimension in the therapy besides the communication between the therapist and the client. The drama and theatre processes have a crucial role in drama therapy interventions and may contribute to therapeutic change.

In the triangular relationship between client, drama, and drama therapist, these means require a continuously adapted approach from the drama therapist to the client. This is in line with three included studies [76,79,82] in which the authors describe the adaptive therapeutic attitude as an open attitude where the drama therapist is constantly attuned to the fun and playfulness from the perception of the client. From there, the drama therapist first creates a variety of opportunities to teach the client how to use drama and play. Subsequently, the drama therapist encourages the children and adolescents to express their wishes for specific roles and personal themes and facilitates playing out personal problems. In parallel, the drama therapist encourages the children and the adolescents to work together and build cohesion and share personal stories [76,79,82]. This is in line with the drama therapy interventions described in the included studies, where drama therapy is provided in groups where there is room to pay attention to individual therapeutic goals. This is confirmed by previous literature and theoretical insights where the drama therapist offers the client the opportunity to explore new roles (including different behavior, feelings and thoughts) within the interactive play by continuously tuning in [42,46,47,49,51,67]. This variety of means combined with adaptive therapeutic attitude is considered an important trigger of mechanism of change in drama therapy [34,46,47,52].

Mechanisms of change are therapeutic processes which arise in the here and now during drama therapy sessions. We found nine mechanisms of change. Regarding these mechanisms, we found three mechanisms of change that arise in common psychotherapeutic processes. These are the psychotherapeutic processes activated by the drama therapeutic means where expression is stimulated, where clients become aware of themselves and others, and where the clients gain more self-control. In addition to common psychotherapeutic processes, we found six mechanisms of change that can be considered as creative arts processes. These are the processes activated by the drama therapeutic means where the clients reflection, creativity, imagination, witnessing, and sharing are stimulated, and where clients gain experiences. Finally, we found two specific drama therapeutic mechanisms of change. These are the processes of embodying a role and expressing emotions of the drama activity. This is in line with previous literature and theoretical insights where these mechanisms of change are considered as the core processes of the drama therapy and creative arts therapies [34,46,47,48,114,115,122,123,124]. All nine mechanisms of change were frequently mentioned in the various studies. That is, the mechanisms were used to focus on different therapeutic targets and treat different psychosocial problems, resulting in a significant change. Therefore, these mechanisms can be considered transdiagnostic mechanisms of change [125].

The aim of this review was to investigate the effects of drama therapy for children and adolescents and to identify the drama therapeutic means, attitude and mechanisms of change that lead to these effects. The conclusions of this review need to be phrased carefully, since the methodological quality of the included studies varied substantially. Only two studies had a strong quality, three studies were rated to have a moderate quality, and five had a low quality. Suboptimal quality was due to measurement instruments that were not investigated for reliability and validity. In addition, some of the studies included a small number of participants. In addition, in one study [78], drama therapy was part of a responsive aggression regulation therapy, and only the whole therapy program was evaluated. Therefore, it is unclear to what extent drama therapy contributed to the effects. Finally, some studies did not show any results on goals that were not the studies’ primary aim. Hence, we need to be careful with conclusions, and more research is imperative. The interventions in the included studies are based on good clinical practice. However, the descriptions of the interventions were brief or described in general terms. No direct relations were drawn between the effects, drama therapeutic attitude, and mechanisms of change. Likewise, the individual means, attitude, and supposed mechanism of change were not empirically investigated in the included studies. Finally, we did not perform a meta-analysis on the effect sizes, because only three of the included studies reported effect sizes. Thus, given these limitations, further research is warranted.

In future research, it is important to make a clear description of the drama therapy intervention, explicating goals and expected effects, and defining the general and drama therapeutic means, therapeutic attitude, and mechanisms of change that are applied. This is not only important for empirical reasons but also for the professionalization of drama therapists. Detailed descriptions allow clinical practice to transfer interventions into common practice among drama therapists as well as to disentangle the effects of specific elements of drama therapy interventions. Future studies should provide detailed descriptions that allow us to relate the drama therapeutic means and therapeutic attitude to the beneficial effects of (different elements of) drama therapy interventions. Moreover, a detailed description of supposed mechanisms of change in drama therapy interventions allows us to investigate why drama therapy may lead to specific effects.

Besides working on clear descriptions of interventions, future studies should investigate designs that fit clinical practice and apply these in a stepwise manner, e.g., starting with single-case experimental designs, feasibility studies, and eventually—when promising—randomized clinical trials. It might be necessary to use a personalized research approach. Personalized research with individual goals and clearly described tailored interventions can give more insights into the effects and how drama therapy contributed to the intervention outcome. The Goal Attainment Scale (GAS) may be considered useful for a more personalized research. This review showed that first steps have been made, where drama therapists explore theoretically how drama therapy influences cognition, emotions, and behavior. It is important to further clarify the relationship between cognition, behavior and emotions and drama therapeutic means, attitude, and working mechanisms to develop a theoretical foundation for further research. For instance, Frydman [59] described the link between the role theory [126,127,128] and executive functioning (EF).

The results of this review provide a starting point to give an overview of the interplay between drama therapy and neuropsychology.

## 5. Conclusions

This study has shown that drama therapy can decrease psychosocial problems in children and adolescents. Our review shows positive effects of the drama therapy intervention on psychosocial problems overall, a decrease in depressive symptoms, (social) anxiety, posttraumatic stress, inattention (especially on hyperactivity and impulsivity), aggressive behavior such as hostility, violent behavior and an increase in assertiveness. In addition, drama therapy had an indirect effect on school behavior, i.e., a positive effect on learning behavior and on school abilities. The drama therapeutic means were applied to create a dramatic reality. The use of the drama therapeutic means was flexible within an adaptive approach. Several mechanisms of change were proposed and partly overlap in different treatments. These mechanisms of change can be considered as transdiagnostic. Overall, descriptions of the means, drama therapeutic attitude, and mechanisms of change in the studies included in this review were described poorly. Therefore, further research is needed to obtain more insight into the effective elements of drama therapy and their mechanisms of change. When we know which and how these elements can contribute to a decrease in psychosocial problems in children and adolescents, then drama therapy can be applied (even) more effectively.

## Figures and Tables

**Table 1:** Formal characteristics of included studies.

First Author/Year	Design/Time Points	Quality Assessment Rate	Study Population	*n* = (Treated/Control)	Type (Group or Individual or Both), Frequency, Duration	Control Intervention/Care as Usual
Anari, 2009 [75]	CCT	Moderate	Age 10–11	14 (7/7)	Group	No intervention
	Follow-up: 3 months		Social anxiety disorder		12 times	
			Elementary school		120 min per session	
					Twice per week	
D’Amico, 2010 [76]	Pre- and post-test design	Moderate	Age 1–12	6	Group	-
			Asperger’s syndrome or High-Functioning Autism and Pervasive Developmental Disorder Not Otherwise		21 sessions	
			Specified Social service center		75 min per session	
					Once per week	
Ghiaci 1980 [77]	CCT	Weak	Age 3–5	12 (6/6)	Individually in a group setting	No intervention
	Follow-up: 1 month		Young children	Follow up: 8 (4/4)	6 sessions	
			Day nursery		60 min per session	
					Six successive weekdays	
Hoogsteder, 2014 [78]	CCT	Weak	Age 16–19	91 (63/28)	Individual and group	Care as usual
			Delinquents (combination of conduct disorder *n* = 30, oppositional disorder *n* = 24, Attention Deficit Hyperactivity Disorder *n* = 11, mental disability *n* = 15)		Average duration in weeks 46.86	
			Secure juvenile justice institution		Average hour of treatment per week 1.72	
					Individual: 60 min, once per week Group: 12–14 sessions 90 min	
Hylton, 2019 [79]	Pre- and post-test design	Moderate	Age 14.71 (mean)	11	Group	-
			Students affected by the February 14th shooting at MSD High School in Parkland Florida		Four days per week over two weeks	
			Summer arts trainings camp		3.5 h for a total of eight sessions (28 h)	
					The two-week camp was held three times, four, five and 5.5 months after the date of the shooting.	
Irwin, 1972 [80]	RCT	Weak	Age 7–8	12 (4/4/4)	Group	Group II: activity psychotherapy group in which regular group social work principles were applied Group III: recreation group in which the workers assumed the role of recreation leaders
			Emotionally disturbed children		20 sessions	
			Outpatient treatment center		60 min per session	
					Once per week	
Lowenstein, 1982 [81]	RCT	Weak	Age 9–16	5	Individual and group	No intervention
			Extreme shyness in maladjusted children		6 months	
			School psychological service			
Mackay, 1987 [82]	Pre- and post-test design	Weak	Age 12–18	5	Group	-
			Girls who have been sexually abused,		8 sessions	
			Special organized location: drama studios at Concordia University in Montreal		4–5 h per session	
					Once per week	
Rousseau, 2007 [83]	RCT	Strong	Age 12–18	123 (66/57)	Group	No intervention
			Newly arrived immigrant and refugee adolescents		9 sessions	
			Integration classes in a multiethnic high school		75 min per session	
					Once per week	
Rousseau, 2012 [84]	RCT	Strong	Age 12–18	55 (27/28)	Group	No intervention
			Immigrant and Refugee		12 sessions	
			High school serving an underprivileged neighborhood of immigrants		90 min per session	
					Once per week	

**Table 2:** Results and description of effects drama therapy intervention.

First Author/Year	Psychosocial Outcome Domain/Measure	Results	Effect Sizes
Anari, 2009 [75]	Self-report Leibowitz social anxiety scale for children and adolescents (LSAS-CA) [85] Performance anxiety subscale Performance avoidance Social anxiety subscale Avoidance subscale	The experimental group showed significant decline in symptoms of social anxiety (all subscales) compared to the control group (*p* < 0.05). The therapeutic changes lasted after three months, and these scores of three months differ from the scores of the control group	No information given
D’Amico, 2010 [76]	Social skills improvement system-rating scales (SSIS-RS) [86] - Social skills (SK) - communication, cooperation, assertion, responsibility, empathy, engagement, self-control Problem behaviors (PB) - externalizing, bulling, hyperactivity/ inattention, internalizing. On the parent form as well as autism spectrum problem behavior	Student Form: The overall mean score on SK and PB did not change significantly after the intervention. There was a significant decrease in the symptoms on the mean score on the subscale hyperactivity/inattention (*p* < 0.05) after the intervention. All other subscales did not change after the intervention Parent Form: There was a significant decrease in the symptoms on the mean score on the overall the SK and PB score (*p* < 0.05) after the intervention. Regarding the subscales, there was a significant decrease after the intervention for externalizing problem behavior, engagement, hyperactivity/inattention, autism spectrum problem behavior (*p* < 0.05). Other subscales did not change after the intervention	No information given
Ghiaci 1980 [77]	Repertory grids * were employed to depict the systems of personal constructs, since these permit a description of an individual’s cognitive structure to be given in his own terms	Compared to the control group, the experimental group showed a larger increase from pretest to posttests on both the original constructs (*p* < 0.025) as well as the focused constructs (*p* < 0.01)	No information given
Hoogsteder, 2014 [78]	Structured assessment of violence risk in youth (SAVRY) [87,88] Three risk domains (1) historical factors (2) social/contextual risk factors (3) individual dynamic risk factors Aggression incidents was based on the data registered by prison staff * Self-control, assertiveness and dealing with anger assessed by juvenile- and mentor report * Self-report Utrecht coping list (UCL) [89,90] Cope with stressful situations: - Problem-focused coping - Palliative coping - Social support - Reassuring thoughts Self-report Brief irrational thoughts inventory (BITI) [91] Measure cognitive distortions on aggression (externalizing) and sub-assertive (internalizing) HIT [92] Self-report on physical aggression and opposition-defiance	All analyses were controlled for pre-test score, gender, length of stay, and participation in EQUIP, a CBT based module Risk of recidivism and aggressive behavior The experimental group had a significant lower violent recidivism risk (*p* < 0.001), higher score on assertiveness (*p* < 0.05 reported by the mentors and *p* < 0.001 reported by the juveniles), lower scores on self-control skills (*p* < 0.001 reported by the mentors and by the juveniles), and on dealing with anger (*p* < 0.001) after the intervention compared to the control group. Fewer incidents were registered in the experimental group, but there was no significant difference Coping skills The experimental group scored significantly better on coping skills problem solving (*p* < 0.001), palliative coping (*p* < 0.001), social support (*p* < 0.001), reassuring thought (*p* < 0.001), and lower scores on stress and poor coping (*p* < 0.001) after the intervention compared to the control group Cognitive distortions Compared to the control group, the experimental group showed significantly lower on aggression/justification (*p* < 0.001), physical aggression (*p* < 0.001), opposite behavior scales (*p* < 0.001), and sub-assertive (*p* < 0.001) after the intervention. There was no significant difference after the intervention on negative attitude Responsiveness The experimental group scored compared to the control group significantly better for motivation for treatment (*p* < 0.05), attention deficits (*p* < 0.05), and scored significantly lower on medium to large for distrust (*p* < 0.001), and impulsivity (*p* < 0.001) after the intervention	SAVRY Recidivism Risk 1.01 Dealing with anger 0.84 AR-list Juv. Self-Control 2.36 Assertiveness 1.99 AR-list mentor Self-Control 1.38 Assertiveness 0.35 UCL Problem Solving 1.37 Palliative Coping 1.73 Social Support 1.05 Reassuring Thought 0.92 SAVRY Stress—Poor Coping 0.49 BITI Aggression/justification 1.38 Sub assertiveness 0.55 HIT Oppositional behavior 0.95 Physical Aggression 1.45 SAVRY Negative Attitude 0.30 SAVRY Motivation for treatment 0.42 Distrust 0.73 Attention deficit 0.45 Impulsivity 0.73
Hylton, 2019 [79]	Depression was measured by self-report Patient Health Questionnaire (PHQ-8) [93] Anxiety was measured by the self-report Generalized anxiety disorder (GAD-7) [94] Posttraumatic stress was assessed using the self-report child’s reaction to traumatic events scale (CRTES) [95] Positive and negative affect were assessed using self-report positive and negative affect schedule (PANAS) [96] Satisfaction of the treatment was assessed using an evaluation questions * especially developed for the camp	The drama treatment program resulted in significant decreases in symptoms of posttraumatic stress (*p* < 0.023), anxiety (*p* < 0.007), depression (*p* < 0.034), and in increases in positive affect (*p* < 0.009). There was no effect on the negative affect after the intervention in the drama group. Participants of the creative arts therapies camp, including visual arts (*n* = 15) music (*n* = 8) and drama (*n* = 11), evaluated: 93.3% agreed or strongly agreed and 6.1% indicating neutrality and 0% disagreed or strongly disagreed on having fun at the camp; 79.8% agreed or strongly agreed and 15.2% indicating neutrality and 6.1% disagreed or strongly disagreed that they learned something new about myself; 84.4% agreed or strongly agreed and 12.5% indicating neutrality and 3.1% disagreed or strongly disagreed that they felt safe at the camp; 87.9% agreed or strongly agreed and 6.1% indicating neutrality and 6.3% disagreed or strongly disagreed that engaging the creative arts gives me a deeper understanding of myself and others	No information given
Irwin, 1972 [80]	Rorschah Index of Repressive Style (RIRS) [97] indicate the extent to which images, emotions and past experiences are verbally labeled and thus available in consciousness in communicable terms Verbal Fluency (VF)—assessing each child’s response to a set of thematic pictures which was designed to elicit projective material through a verbal modality Semantic Differential (SD) * – specifically designed to measure attitude changes: three dimensions: evaluative, potency, activity. Each had six concepts (me, grown-ups, feelings, sharing, imagination, other kids) Parent Competence Scale (PCS) *—to measure mastery of major areas of functioning both at home and with peers and consisted of concrete descriptions of child behavior: Factor I perception degree of interest and participation in activities vs. degree withdrawal and associated depression. Factor II perception of relative degree cooperation and compliance compared to child’s anger and defiance in daily interpersonal relationships	Comparing the change scores, the intervention group showed more positive changes from pre- to posttest in RIRS score (*p* < 0.05) and verbal fluency (*p* < 0.01) compared to the control groups. In addition, change scores between pre- to post were significantly higher in the intervention group compared to the control groups on two of the three semantic dimensions of the SDC, namely “evaluating” (Me and Other kids; *p* < 0.05), and “potency” (Me, Other kids and Grown-up; *p* < 0.05). There were no significant differences in either the activity or recreation group after the intervention. From the parent competence scale: Factor I and of factor II rating score differences yielded no significant results for all groups after the interventions	No information given
Lowenstein, 1982 [81]	Maudsley Personality Inventory self-report scale [98] Timidity scale on a 1–5 rating scale, 1 = very timidity, 5 = moderately outgoing Assessed in reading, spelling, and mathematics.	The experimental group had a significantly less severe timidity score (*p* < 0.01) after the intervention compared to the control group. In addition, there was a significant difference changed in intelligence (*p* < 0.05) ** between the groups after the intervention. No differences between groups were seen in attainments in reading, spelling and mathematics after the intervention	Severity of timidity: 2.075 MPI extraversion: 0.998
Mackay, 1987 [82]	Beck depression Inventory (BDI) [99] self-report scale to assess depression level SCL-90 self-report [100] depression, anxiety, somatization, interpersonal sensitivity, obsessive-compulsiveness, hostility, phobic anxiety, paranoid ideation and psychoticism Texas social behavior inventory-self-report short form (TSBI) [101] to assess self-esteem Attributional Style Questionnaire (ASQ) self-report [102] attributions were assessed along three dimensions: internal-external, stable-unstable, global-specific Social support questionnaire (SSQ) self-report [103] assess number of social supports and satisfaction with level of social support The Marlowe–Crowne Social Desirability Scale (MCSDS) self-report [104] employed to assess the tendency of the participants to seek social approval by responding in a culturally appropriate manner.	The experimental group showed significant reductions on the levels of hostility (*p* < 0.01), depression (*p* < 0.10), and psychotic thinking (*p* < 0.10) after the intervention. No significant changes between pre- and posttest were found on self-esteem level (TSBI), attribution style (ASQ), number of social supports or reported satisfaction with social supports (SSQ), or social desirability score (MCSDS)	SCL90 Overall intensity of symptoms 1.042 Hostility 0.642 Depression 1.813 Psychoticism 0.561 Anxiety 0.492 Interpersonal sensitivity 0.795 Paranoid ideation 0.345 Obsessive compulsive 0.562 Phobic anxiety0.688 Somatization 0.574 Beck Depression Inventory 1.022 Self-esteem (TSBI) 0.603 Attributional style questionnaire Internal, stable. Global Attributions: bad events 0.309 good events 0.308 Social support questionnaire Number of social supports 0.374 Satisfaction with social supports 0.135 Marlowe-Crowne Social Desirability Scale 0.037
Rousseau, 2007 [83]	Strengths and Difficulties Questionnaire (SDQ) [105]: Emotional and behavioral symptoms Impairment perception: Self-report: Difficulties distress me Interfere with home life Interfere with friendships Interfere with classroom learning Interfere with leisure activities Teacher’s report: Difficulties Distress adolescent Interfere with friendships Interfere with classroom learning Self-Esteem Scale (SES) [106] School performance was assessed on the basis of the first and the last report cards of the school year *	There were no significant differences on emotional and behavioral symptoms at post between both groups, controlling for group differences at baseline The participants in the experimental group reported less impact in all categories except learning at posttest, whereas those in the control group reported more impact on distress (*p* < 0.022) impairment of friendships (*p* < 0.033), and a higher total impact score (*p* < 0.035). No significant group differences were found in the teachers’ reports of the impact scores. Girls in the experimental group showed a significant decrease in the total impact score (*p* < 0.001), whereas boys in the control group showed a significant increase in the total impact score (*p* < 0.028). No age effect was observed School performance comparing the first and last report cards of the school year showed a significant difference in oral expression (*p* < 0.000) for the experimental group and (*p* < 0.001) for the control group and a significant improvement in mathematics (*p* < 0.005) for the experimental group. Controlling for group differences at baseline, results showed posttest differences between both groups in mathematics. No significant improvement was reported between the first and the last report cards with regard to overall French results of both groups. With regard to self-esteem, the analysis did not show significant differences within groups between pre and post assessment	No information given
Rousseau, 2012 [84]	Strength and difficulty questionnaire (SDQ) self-report [103]	Total SDQ symptom score did not change after the intervention on both, experimental and control, groups. The students of experimental group showed significant decrease in the impact on the impairment (*p* < 0.021) after the intervention. The symptom score of the subgroup of youth who did not report difficulties in school in the countries of origin also decreased following the intervention but not significance (*p* < 0.053)	No information given

* Measurement developed by researchers. ** Results cannot be traced in the study.

**Table 3:** Characteristics of drama therapy interventions.

First Author/Year	Goal of the Study	Intervention	Therapist Attitude	Drama Therapeutic Means and *Supposed Mechanisms of Change of the Intervention*
Anari, 2009 [75]	This study examines the effectiveness of drama therapy in reducing symptoms of social anxiety disorder in children	Emunah’s Integrative Five-phase Model [107]: Focusing on group play and direct teaching of social interactions	No information given	**Participation in a drama activity such as storytelling, movement, voice, role play, pantomime** *Experience positive human relations* *Experience and recreate life situations and actualities*
D’Amico, 2010 [76]	To determine the efficacy of drama therapy in addressing the children’s performance or acquisition deficits across the social skill domains targeted over the course of the project (determined by the results obtains on the SSIS-RS forms)	The weekly sessions using each skill from the SSIS as a theme for the two subsequent weeks. Therapeutic modality based on the child’s social and behavioral needs The drama therapy techniques centered on making connections among the group members, while discovering commonalities and shared interests, and encouraged self-expression. Used components of drama therapy: dramatic projection; dramatic reality; role-playing; and storytelling	Adaptive approach	**Dramatic projection through improvisational scenes** *Express their own ideas* *Emotional expression* **Dramatic reality within a playspace using improvisational scenes with both conflict and cooperative activities where children act out different social issues.** *Creativity* *Experiencing (social connection)* *Explore their vulnerabilities and psychological issues and reflection on experiences, feelings, and emotions of oneself and others* **Role-playing** *Explore new identities* *Embody the personas* *Share experiences and feelings* *Observing (non-verbal) behavior and interpreting behavior of others* **Storytelling** *Expression of experiences, feelings, emotions, and thoughts* *Reflection on experiences, feelings, and emotions of oneself and others* *Self-control, participants become active participants in their own treatment* **General** *Fun and playfulness* *Use imagination*
Ghiaci 1980 [77]	Cognitive change	Each session comprised five stages: act out an event individually in a group setting children divided themselves into pairs and carried out a cooperative activity children divided themselves into groups of three and performed children divided themselves into two groups and enacted a short piece of drama relaxation individually enactment in a group setting	No information given	No information given
Hoogsteder, 2014 [78]	Decrease severe aggressive behavior	Re-ART: a cognitive behavioral approach combined with drama therapeutic techniques, role-playing games in order to practice perspective taking and problem solving skills. All arts therapists targeted self-image, emotions, and social interaction (especially situations that elicit aggressive behavior), but they did not use any form of established manualized treatment	No information given	**Role-playing games** *Perspective taking*
Hylton, 2019 [79]	Improving mental health status by decreasing symptoms of PTSD, depression levels, anxiety levels and lower levels of negative affect and by increasing positive affect. Drama therapy Role theory and method: participants explore life roles in order to gain insight into group dynamics and internalize new roles that help expand individual resilience and strengths	Improvisation exercises: Participants activate imagination, try new roles, and explore spontaneity. Participants share and enact a personal story with group members in order to promote empathy, insight, and interpersonal connection. Projective technique: each participant chooses and object that he/she feels connected to and verbalizes how he/she feels through the use of this projective	The therapist gave the participants the freedom to share the traumatic memory however they felt comfortable	**Improvisation exercises to imaginal exposure, explore life roles and acting out stories through bodily and verbal processing** *Explore life roles* *Reflection on experiences, feelings, and emotions of oneself and others* *Embodied emotional experience* *Share experiences, feelings, and emotions of oneself and others* *Activate imagination* *Explore spontaneity* *Internalize new roles* **Projective technique** *Emotional expression* *Verbal expression* *Reflection on experiences, feelings, and emotions of oneself*
Irwin, 1972 [80]	Exploring the feasibility of using drama therapy as a form of treatment with emotionally disturbed children. Prepare inarticulate non-communicative children emotionally for more traditional forms of verbal psychotherapy by learning a progressive sequence of communication skills through dramatic play	Improvisational dramatic play to express and play out wishes, conflicts and fantasies	No information given	**Repeated experiences in improvisational dramatic play** *Share feelings* *Making emotional discrimination* **Play out** *Share feelings* *Witnessing* *Immediate feedback and reflection on experiences, feelings, and emotions of oneself and others* *Express internal states in verbal terms* **Playing a role** *Expression in a role*: - *Verbal expression* - *Nonverbal expression*
Lowenstein, 1982 [81]	Treat the problem of timidity by reducing anxiety, increasing assertiveness, promoting the ability to communicate effectively with other people, treating feelings of inadequate, influencing parental background and decreasing over-sensitivity	Drama therapy, in which timid children were given especially extroverted and assertive parts in contrast to their normal introverted or non-assertive demeanor.	No information given	No information given
Mackay, 1987 [82]	A primary goal of the program, structured drama therapy, was to help establish feelings of power and control to combat the feelings of worthlessness and loss of integrity and power often associated with rape and incest	Improvisation, roleplaying and storytelling	The views of Carl Rogers where expression of self is best fostered in an atmosphere of psychological safety	**Symbolic role playing (as a projective technique)** **Improvisation** **Storytelling** *Expression of feelings, thoughts, and their identity* *Creativity* *Share thoughts or experiences* *Experience*: - *Fun and playfulness* - *of acceptance and being heard* - *of getting close to each other* - *acting out ideas and feelings* - *control in their role play*
Rousseau, 2007 [83]	The goal of the drama therapy program was to give young immigrants and refugees a chance to reappropriate and share group stories, in order to support the construction of meaning and identity in their personal stories and establish a bridge between the past and present	The program is based in Augusto Boal’s forum [108] and Jonathan Fox’s playback theater [109]	No information given	**Pairs technique** *Reflect on a person’s contradictory feelings* *Reflect different points of view of the same situation or experience* **Storytelling, acting** *Exploration of ideas and feelings associated with key experiences* *Sharing strong emotions and subsequent relief* *Feeling of agency* **Symbolic play** *Expression* *Witnessing others*
Rousseau, 2012 [84]	The goal is to alleviate problems associated with distress, behaviors stemming from the losses of migration and the tensions of belonging to a minority in the host society, as well as to improve social adjustment, academic performance, and to provide schools and teachers with tools for adapting their teaching methods to suit the emotional and social needs	Each session includes a warm-up period composed of theatrical exercises and of a language awareness activity which also uses dramatization	No information given	**Theatrical exercises, dramatization, play out stories** *Sharing of stories* *Creation of links among participants*

**Table 4:** Quality of the studies.

First Author/Year	A. Selection Bias	B. Study Design	C. Confounders	D. Blinding	E. Data Selection Methods	F. Withdrawals and Dropouts	Overall
Anari, 2009 [75]	Moderate	Strong	Weak	Moderate	Strong	Strong	Moderate
D’Amico, 2010 [76]	Moderate	Moderate	Weak	Weak	Strong	Strong	Moderate
Ghiaci 1980 [77]	Weak	Moderate	Weak	Weak	Weak	Weak	Weak
Hoogsteder, 2014 [78]	Moderate	Moderate	Weak	Weak	Moderate	Moderate	Weak
Hylton, 2019 [79]	Moderate	Moderate	Strong	Moderate	Weak	Moderate	Moderate
Irwin, 1972 [80]	Weak	Moderate	Weak	Moderate	Weak	Weak	Weak
Lowenstein, 1982 [81]	Moderate	Strong	Weak	Weak	Strong	Strong	Weak
Mackay, 1987 [82]	Moderate	Moderate	Weak	Weak	Strong	Strong	Weak
Rousseau, 2007 [83]	Moderate	Moderate	Strong	Moderate	Strong	Strong	Strong
Rousseau, 2012 [84]	Moderate	Strong	Moderate	Moderate	Strong	Strong	Strong

## Data Availability

Not applicable.
